# Exploring the Potential of Wild Andean Blueberries for Powdered Juice Production through Spray Drying

**DOI:** 10.3390/foods12122348

**Published:** 2023-06-12

**Authors:** Mauren Estupiñan-Amaya, Carlos Alberto Fuenmayor, Alex López-Córdoba

**Affiliations:** 1 Grupo de Investigación en Bioeconomía y Sostenibilidad Agroalimentaria, Escuela de Administración de Empresas Agropecuarias, Facultad Seccional Duitama, Universidad Pedagógica y Tecnológica de Colombia, Carrera 18 con Calle 22, Duitama 150461, Colombia; mestupinana@unal.edu.co; 2 Instituto de Ciencia y Tecnología de Alimentos (ICTA), Universidad Nacional de Colombia, Av. Carrera 30 # 45-03, Bogotá 111321, Colombia; cafuenmayorb@unal.edu.co

**Keywords:** bioactive compounds, food powders, encapsulation, food ingredients, wild fruits

## Abstract

The Andean blueberry (*Vaccinium meridionale* Sw) is an underutilized wild fruit native to South America. It is known for its antioxidant properties and potential health benefits. In this study, Andean blueberry juice powders were produced via spray drying, using maltodextrin (MD), gum Arabic (GA) or a combination of both (MD:GA) as wall materials. The spray-dried juices were analyzed for the recovery percentage of total polyphenols and monomeric anthocyanins, as well as for their physicochemical and technological properties. Results showed that the type of carrier agent used caused statistically significant differences in the bioactive content and the antioxidant activity of the powders (*p* < 0.05). It was found that the MD samples has the highest monomeric anthocyanins content (0.88 ± 0.02 mg cyanidin 3-glucoside equivalents/g) and the highest anthocyanins recovery (96.3 ± 1.7%), while the MD:GA powders showed the highest values of total polyphenol content (5.70 ± 0.09 mg gallic acid equivalents/g), DPPH scavenging capacity (2.49 ± 0.02 mg gallic acid equivalents/g) and phenolics recovery (87.2 ± 1.1%). Furthermore, all the spray-dried powders exhibited low moisture content (<5.5%) and water activity (<0.40), as well as high solubility in water (>94%) and good flowability. Future prospects include evaluating the stability of the Andean blueberry juice powders during storage and exploring the formulation of new foods and beverages that incorporate these spray-dried powders.

## 1. Introduction

The growing demand for healthier food choices has driven the worldwide fruit juice market, which includes a diverse range of products such as fruit juice, nectars, powdered juice, and others [1]. In particular, there has been a significant increase in interest in powdered juice due to its convenience and high durability [2]. Additionally, food powders facilitate shipping operations and make them more profitable due to their lower volume and weight, and easier handling [3].

Wild Andean fruits are highly appealing in the fruit juice market due to their unique and exotic flavors, making them a popular choice for consumers seeking new and exciting taste experiences [4,5]. Moreover, many consumers are willing to pay a premium for wild Andean fruits, because they are perceived as being healthier than their cultivated counterparts, as they are often grown without the use of pesticides or other chemicals [4,6].

The Andean blueberry *(Vaccinium meridionale* Swartz) is a wild shrub that grows in South America at altitudes of over 2300 m above sea level (m.a.s.l.). Its fruit is widely recognized for its high content of bioactive compounds, including anthocyanins, flavonoids, and phenolic acids, which have been associated with various health benefits [7,8]. Despite this, Andean blueberry fruits are underutilized and not commonly consumed. There is a limited supply of value-added products, which contributes to the lack of popularity of this fruit.

Few studies have been conducted on the development of Andean blueberry juice powders. Estupiñan et al. studied the effect of using different concentrations of maltodextrin and their mixtures with gum Arabic on the efficiency of the encapsulation of the bioactive compounds, physicochemical characteristics, and technological properties of Andean blueberry juice powder obtained via freeze-drying. The results showed that the powders obtained had a polyphenol recovery rate of over 64%, a monomeric anthocyanin recovery rate of over 66%, low water activity (<0.5), high solubility (>91%), and good flow properties (angle of repose < 37°) [9,10].

Spray-drying technology is widely used in the food industry to produce powdered products, such as milk, flavors, instant drinks, soups, tea, and eggs, among others [11,12]. This technology has the advantage of being economical, scalable, and versatile, and allows for continuous operations [13,14]. Maltodextrin, a GRAS ingredient approved by the FDA, is the most used encapsulating material in the industry for obtaining powdered products through spray drying because it has good film-forming properties, low hygroscopicity, and good solubility [15]. However, some studies have proposed the use of mixtures of maltodextrin with other materials such as gum Arabic to achieve higher efficiencies in retaining bioactive compounds [15,16]. It has been suggested that the addition of gum Arabic improves the emulsification and film-forming properties of the mixtures because of its low protein content [16]. Navarro-Flores et al. evaluated different combinations of wall materials to produce chipilin (*Crotalaria longirostrata*) extract powders using spray drying, finding that a combination of maltodextrin and gum Arabic allowed for an increase in the spray-drying yield and the retention of higher amounts of phenolic compounds in the powders [17]. Gácina et al. spray dried blackthorn flower extract using maltodextrin or its combination with gum Arabic or inulin and found that the highest concentrations of total hydroxycinnamic acids, total flavanols, total flavones, and total phenolic content were achieved when maltodextrin/gum Arabic mixtures (75:25) were used [16].

Recent developments in the spray drying of other berry juices such as the blueberry, bilberry, blackberry, chokeberry, and maqui berry have been reported [11,14]. Encapsulating agents, such as maltodextrin, gum Arabic, or a combination of both, are commonly used in these studies [18,19,20]. Additionally, other materials such as hi-maize, carboxymethyl cellulose, whey protein concentrate, and soy protein isolate have also been explored [21,22]. The physicochemical and technological properties of spray-dried fruit juice powders are known to be closely linked to the formulation of the liquid feed (e.g., feed composition, concentration and type of carrier agent, and viscosity) and the operating conditions of the spray-drying process (e.g., inlet and outlet temperatures, flow rate, and atomizer type) [11,14]. For instance, Bastías-Montes et al. studied the physicochemical characteristics of maqui extract powders obtained through spray drying using different inlet air temperatures (150 °C, 160 °C, and 170 °C). Their findings indicated that powders produced at 170 °C exhibited several favorable characteristics, including lower moisture content (1.61%), reduced water activity (0.15), the minimal loss of total polyphenol content (23.05 mg GAE/g), and higher solubility (92.70%) [23]. Recently, Gawałek investigated the effect of different inlet air temperatures (150–185 °C) and feed solution concentrations (15–45% d.m.) on various aspects of the spray-drying process of chokeberry juice, including the powder yield, energy consumption, and bioactive properties of the resulting powders [12]. The results showed that the highest levels of total polyphenols and anthocyanins were achieved when using an inlet air temperature of 150 °C and feed solution concentrations ranging between 25% and 30% of dry matter. However, the optimal process parameters for minimizing specific energy consumption were found to be an inlet air temperature range of 160 °C to 170 °C and a feed solution concentration range of 30% to 35% dry matter.

To date, limited research has focused on the spray-drying process of Andean blueberry juice. Therefore, this study aims to investigate the use of commonly employed encapsulating materials (maltodextrin, gum Arabic, and the combination of both) to produce Andean blueberry powders and evaluate their resulting bioactive contents, antioxidant activity, physicochemical properties, and technological characteristics. It has been hypothesized that the choice of carrier agents in the spray-drying process significantly affects the bioactive content, antioxidant activity, and overall properties of Andean blueberry juice powders.

## 2. Materials and Methods

### 2.1. Materials

Gum Arabic (GA) and maltodextrin (MD) with a dextrose equivalent (DE) 18–22 derived from maize starch were used as carrier agents. Both materials were provided by Tecnas S.A. (Medellín, Colombia).

Ripe Andean blueberries from Ráquira (Boyacá, Colombia) were used. The fruits were selected, sorted, washed, and disinfected using 100 mg L^−1^ hypochlorite solution.

Andean blueberry juice was extracted using a juice press to squeeze the crushed fruits and then filtered through Whatman paper N°1 under vacuum. The properties of the resulting juice were reported in a previous work (dry solid content = 11.6% and soluble solid content = 13.3 °Brix) [9].

### 2.2. Spray-Drying Process

The preparations for spray drying were carried out by mixing 80 wt.% of Andean blueberry juice with 20 wt.% of either maltodextrin (MD), gum Arabic (GA), or a blend of both at a 1:1 ratio (MD:GA). These ratios were chosen based on preliminary experimental results and previous works [12,19].

All encapsulating agents were dissolved in the juice under constant stirring at 800 rpm (EUROSTAR 20 vertical agitator, IKA, Staufen, Germany). The MD, GA, and MD:GA formulations resulted in dry matter contents of around 30%.

The operational conditions of spray drying were chosen based on previous works [19,24]. The homogenized blends were fed into a BUCHI B-290 mini spray dryer (Flawil Suiza) at a feed flow rate of 8 mL/min. The inlet temperature was 170 °C, and the outlet air temperature was 80 °C. The inlet air flow was 32 m^3^/h and the compressed air caudal was 414 L/h. The obtained samples were stored in a hermetic flask until use.

In preliminary experiments, the spray drying of juices without encapsulating agents was also carried out, but the juice adhered to the walls of the spray cylinder, so no powders were obtained.

### 2.3. Charactetization of the Spray-Dried Andean Blueberry Juice Powders

#### 2.3.1. Color Attributes

The color of the samples was assessed using a colorimeter (Konica-Minolta CR-10, Osaka, Japan), which provided color values in the CIELab system (L* lightness, a* redness/greenness, and b* yellowness/blueness). Hue angle (*h*) and chroma were determined using Equations (1) and (2), respectively:

(1) $$h=\tan^{-1}\left(\frac{b*}{a*}\right)$$

(2) $$Chroma={\left[{a*}^{2}+{b*}^{2}\right]}^{1/2}$$

#### 2.3.2. Scanning Electron Microscopy Analysis

The spray-dried powders were examined using a ZEISS EVO MA10 microscope (Carl Zeiss SMT Ltd., Cambridge, UK). The samples were attached to stubs, sputter layered with gold, and observed under an acceleration voltage of 20 kV. The particle size of the powders was estimated via SEM micrograph analysis using image processing software (Image J, NIST, Bethesda, Rockville, MD, USA).

#### 2.3.3. Fourier Transform Infrared Spectroscopy (FTIR)

FTIR spectra were obtained using a FT/IR-4100 unit (JASCO, Hachioji, Tokyo, Japan) equipped with an attenuated total reflectance (ATR) accessory, averaging 24 scans between 4000 and 450 cm^−1^, at a resolution of 4 cm^−1^.

#### 2.3.4. Recovery of Total Polyphenolic Compounds and Total Monomeric Anthocyanins

The total polyphenol content of both the Andean blueberry juice and powders was measured as reported in previous work [9,25]. Briefly, 400 µL of a sample was blended with 2 mL of Folin–Ciocalteu reagent (Panreac, Barcelona, Spain) diluted 10 times. Subsequently, 1.6 mL of sodium carbonate (7 g/100 mL) was added to each blend, and after 30 min of reaction time, the absorbance was recorded at 760 nm using a spectrophotometer (X-ma 1200 Human Corporation, Loughborough, UK). A calibration curve was prepared using gallic acid (Merck, Darmstadt, Germany) as a standard, and the results were expressed as gallic acid equivalents (GAE) per gram of dry solids (mg GAE g^−1^).

To determine the total content of monomeric anthocyanins, the pH differential method was used [26]. The absorbances were read at 520 and 700 nm using the spectrophotometer, and the concentration of anthocyanins was determined and expressed as cyanidin 3-glucoside equivalents (C3G) using an extinction coefficient (ε) of 26,900 L cm^−1^ mol^−1^ and a molecular weight of 449.2 g. mol^−1^.

The results of the loading capacity were expressed as GAE per gram of dry solids (mg GAE g^−1^) and C3G per gram of dry solids for total polyphenols and monomeric anthocyanins, respectively.

The recovery percentages of total polyphenolic compounds and total monomeric anthocyanins after spray drying were obtained using Equation (3):

(3) $$Recovery\left(\%\right)=\frac{L_{c}}{L_{0}}\times 100$$

where L_c_ corresponds to concentration of total phenolic compounds or monomeric anthocyanins in the reconstituted spray–dried powders and L_0_ is the concentration of total phenolic compounds or monomeric anthocyanins of the dispersion fed to the spray dryer.

#### 2.3.5. DPPH^•^ Radical Scavenging Activity

The DPPH^•^ free radical method was used to evaluate the antioxidant activity of the spray-dried powders [27]. Briefly, 100 µL of each reconstituted juice was blended with 3.9 mL of a 25 mg. L^−1^ ethanolic solution of the 2,2-diphenyl-1-picrylhydrazyl (DPPH^•^) reagent (Sigma-Aldrich, St. Louis, MO, USA). The blend was then left to react until the plateau phase was reached and the absorbance was recorded at 515 nm. A calibration curve was prepared using GA as a standard, and the results were expressed as mg GAE g^−1^.

#### 2.3.6. Moisture Content and Water Activity

The moisture content (%) of the spray-dried samples was determined gravimetrically by drying the samples in an oven at 105 °C until a constant weight was obtained. Water activity (a_w_) values were measured using an AquaLab Serie 3 TE apparatus (Pullman, WA, USA).

#### 2.3.7. Water Solubility

The water solubility of the powders was measured by reconstituting 1 g of sample in 100 mL of distilled water under continuous stirring at 1000 rpm for 5 min (IKA RT5 magnetic stirring, Staufen, Germany). The reconstituted juices were centrifuged at 1500 rpm for 5 min, and the supernatant was dried at 105 °C until a constant weight was achieved.

The solubility in water of the samples was calculated as the ratio between weight of dry solids in supernatant and the weight of the dry powder reconstituted in water.

#### 2.3.8. Densities and Flow Properties

The loose bulk density of the spray-dried powders was measured by freely dropping a known sample amount (~1.5 g) into a 25 mL graduated cylinder and was calculated as the mass-to-volume ratio. The tapped bulk density was calculated as the ratio between the sample mass and the volume occupied by it in the cylinder after being tapped manually until a stable value was obtained. The Hausner ratio and Carr index were determined, as described in previous works [9,10].

To determine the angle of repose, a known amount of the sample was poured through a funnel located at a fixed height and allowed to fall freely onto a flat surface. The height (h) and radius (r) of the resulting conical pile were measured, and the tangent of the angle of repose was calculates as the h/r ratio [9,10].

### 2.4. Statistical Analysis

Statistical analysis was carried out using Minitab v.16 software (State College, PA, USA). Analysis of variance (ANOVA) and Tukey’s pairwise comparisons were conducted at a 95% confidence level. All experiments were performed at least three times, and the data were expressed as means ± standard deviations.

## 3. Results

### 3.1. Appereance and Chromatic Attributes of the Spray-Dried Powders

**Figure 1:**
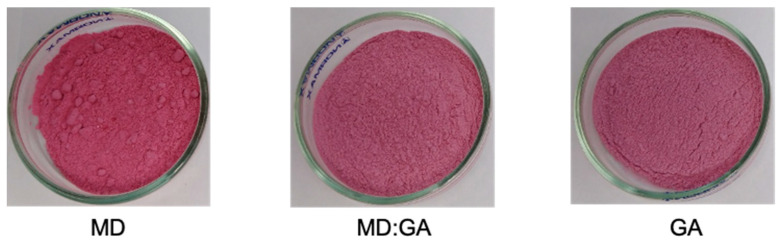
Images of the spray-dried Andean blueberry juice powders produced with maltodextrin (MD), gum Arabic (GA), or a combination of both materials (MD:GA).

Figure 1 shows images of the spray-dried Andean blueberry juice powders produced with maltodextrin (MD), gum Arabic (GA), or a combination of both materials (MD:GA). The resulting powders had a fine and homogeneous appearance with a characteristic color (Table 1). Both MD:GA and GA powders showed a homogeneous appearance (without agglomeration), while some particles adhering to each other were observed in the MD samples.

The lightness (L*) of the GA powders was higher than that of MD powders (*p* < 0.05), while GA and MD:GA powders showed similarities in terms of this characteristic (*p* > 0.05) (Table 1). This behavior can be attributed to the difference in color of the MD and GA materials that makes up the powders. Other authors have reported that the color of gum Arabic is responsible for a higher brightness in encapsulated products [28]. In addition, MD and MD:GA powders showed higher a* values than GA powders, displaying a more reddish color. No significant differences were found between the a* values of the MD and MD:GA samples. Furthermore, MD and MD:GA powders showed higher chroma and *h* values than GA powders, indicating that the use of maltodextrin alone or mixed with gum Arabic allowed for the better preservation of powder color compared to gum Arabic. Similar results were reported by Pieczykolan and Kurek ([29] and Silva et al. [30]).

**Figure 2:**
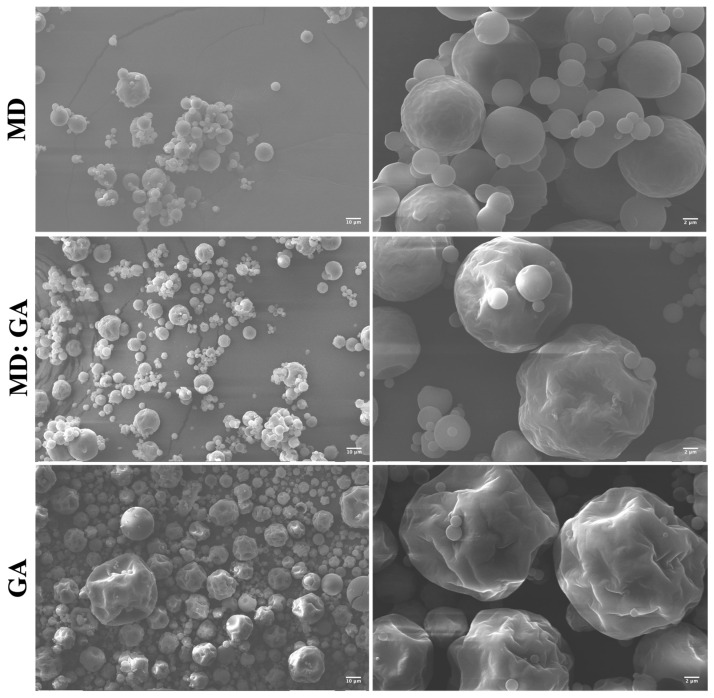
SEM images of the spray-dried Andean blueberry juice powders produced with maltodextrin (MD), gum Arabic (GA), or a combination of both materials (MD:GA).

Figure 2 shows the SEM images of the MD, GA, and MD:GA spray-dried powders. The use of maltodextrin as the encapsulating agent resulted in the formation of spherical particles with a smooth and homogeneous surface (Figure 2). It has been reported that maltodextrin, due to its high content of low molecular weight sugars, can act as a plasticizer, preventing particle surface shrinkage during the spray-drying process [20,31]. In contrast, GA and MD:GA powders exhibited irregular surfaces with some indentations (Figure 2). The formation of these dents on the particle surface during spray drying is a complex phenomenon influenced by several factors [32]. Of these, the drastic loss of moisture during drying followed by cooling plays a key role in inducing particle shrinkage [33]. Furthermore, the concentration and the physical properties of the wall materials, as well as the drying conditions (e.g., drying rate and feed flow), have been identified as factors than can impact the morphology of spray-dried particles [32].

**Figure 3:**
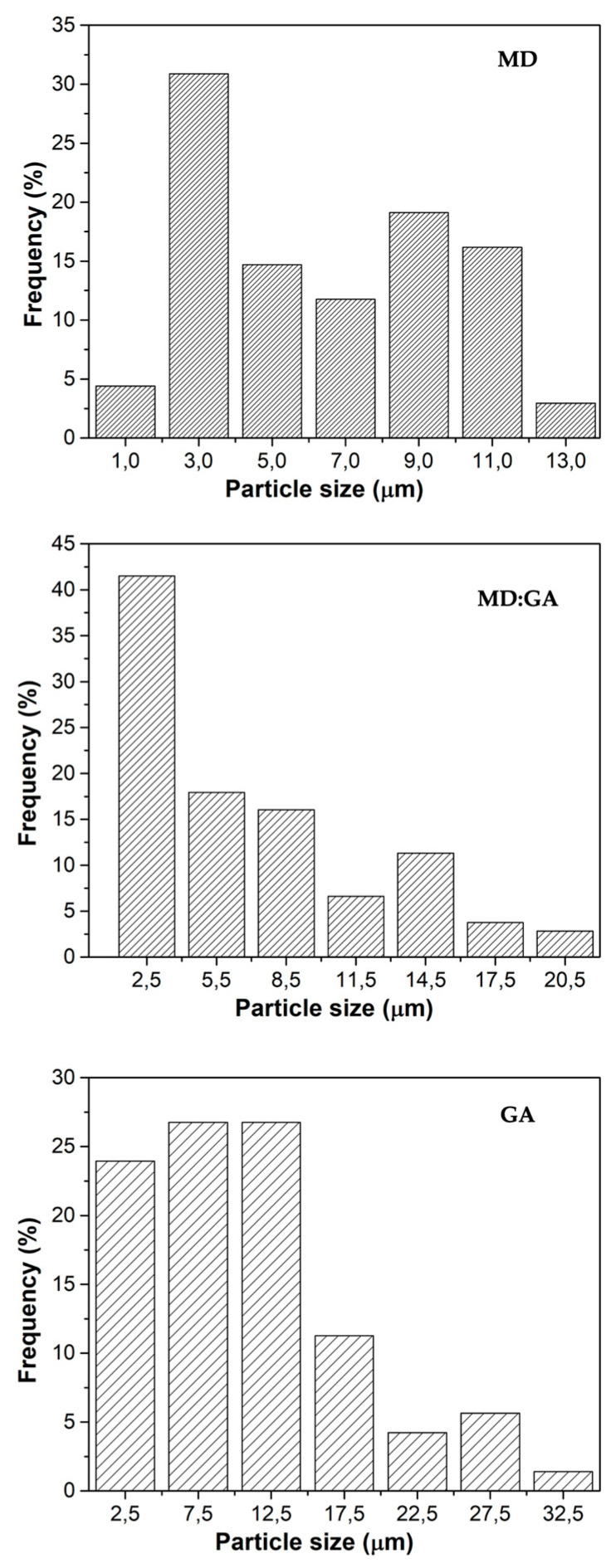
Particle size distribution of the spray-dried Andean blueberry juice powders produced with maltodextrin (MD), gum Arabic (GA), or a combination of both materials (MD:GA).

Particle size is a critical property that plays a significant role in the behavior of powders. This parameter has a direct impact on various powder characteristics, such as dissolution rate, flowability, hygroscopicity, and stability during storage [34]. Figure 3 shows the particle size distribution of the MD, GA, and MD:GA spray-dried powders. It has been observed that the GA particles showed a broader distribution than the MD and MD:GA ones, suggesting that the particle size was relatively more homogeneous in the latter particles. MD and MD:GA samples showed similar average diameters (MD: 6.5 ± 3.3 μm and MD:GA: 7.09 ± 5.3 μm), while the GA powders showed higher mean diameters (11.09 ± 7.5 μm), compared to the other two. Previous studies have reported that gum Arabic has a higher molecular weight and thickening properties compared to maltodextrin, due to its long and complex structure [35,36]. Thus, the larger particle size could be due to the higher viscosity of gum Arabic, which causes the droplets to be more viscous and less deformable, leading to the formation of larger particles during the spray-drying process.

### 3.2. Infrared Spectra of the Spray Dried-Powders

**Figure 4:**
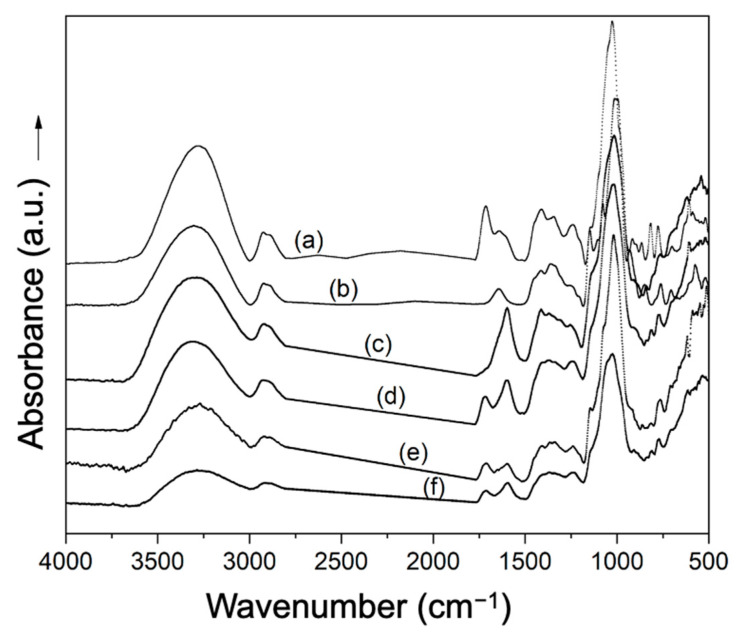
FTIR spectra of (a) Andean blueberry juice; (b) maltodextrin; (c) gum Arabic; and spray-dried Andean blueberry juice powders with maltodextrin (d), gum Arabic, (e) and maltodextrin and gum Arabic (f).

Infrared spectra of MD, GA, and MD:GA spray-dried powders are shown in Figure 4. In addition, the spectra of Andean blueberry juice, maltodextrin, and gum Arabic are shown for comparison. The spectra of MD, MD:GA, and GA powders showed the characteristic signals of each component of the formulation. In the case of MD and MD:GA powders, the characteristic signals of maltodextrin were detected at 3300 cm^−1^ (O–H group stretching), 2905 cm^−1^ (asymmetric C–H_2_ group stretching), 1641 cm^−1^ (free carboxyl groups), 1150 cm^−1^ (C–O group stretching), 1005 cm^−1^ (C–O group stretching), and 929 cm^−1^ (glycosidic bond C–O–C stretching; CH_2_ out-of-plane bending) [37,38]. Additionally, MD:GA and GA powders showed characteristic signals of gum Arabic at 3429 cm^−1^ (–OH stretching), 2930 cm^−1^ (free carboxyl groups), 1590 cm^−1^ (C=O stretching), and 1039 cm^−1^ (C–O stretching) [39,40]. Furthermore, in all powders, the characteristic bands of Andean blueberry juice were observed at 3298 cm^−1^ (OH stretching), 1712 cm^−1^, 1630 cm^−1^, and 1521 cm^−1^ (C=C stretching), and 1024 cm^−1^ (C–O stretching) [41].

### 3.3. Content of Bioactive Compounds and Antioxidant Activity of the Spray-Dried Powders

Table 2 shows the content and recovery percentage of total polyphenols and total monomeric anthocyanins, as well as the DPPH scavenging activity of the MD, GA, and MD:GA spray-dried powders. All samples showed recovery percentages of phenolic compounds higher than 80%, with the highest retention of these compounds in MD:GA powders (*p* < 0.05). The MD powders showed a higher total phenolic content than the GA ones. In addition, it was observed that the MD:GA mixture enabled the production of powders with a higher phenolic compound content compared to MD and GA samples. Similar observations were made by Gácina et al. [16], who encapsulated total polyphenols from blackthorn flower extract using maltodextrin or its combination with gum Arabic or inulin. The better retention of total hydroxycinnamic acids, total flavanols, total flavones, and total phenolic content was achieved with the MD:GA blend (75:25) than when MD was used alone or in combination with inulin.

On the other hand, differences were observed in the content of monomeric anthocyanins of MD, MD:GA, and GA powders (Table 2). GA and MD:GA powders showed similar recovery percentages of anthocyanins, while in MD powders, the retention of anthocyanins increased by 18%. These results were similar to those of Ballesteros et al. (2017), who reported that the use of maltodextrin allowed the production of powders with higher recovery percentages of coffee flavonoids (73%), compared to the use of gum Arabic alone or combination with maltodextrin.

The MD powders showed a higher anthocyanin content than the GA samples, while the MD:GA samples showed intermediate anthocyanin contents between GA and MD. A similar behavior was observed by Pieczykolan and Kurek [29]. These results agree with those obtained through color analysis (Table 1) and with what has been reported by other authors [39,42,43], suggesting that powders with a higher anthocyanin content exhibit higher values in the a* coordinate and hue angle (*h*).

Regarding antioxidant activity, MD powders showed higher DPPH^•^ scavenging activity than GA powders. In addition, it was observed that the MD:GA mixture allowed the obtaining of powders with higher antioxidant activity compared to the MD and GA samples. This behavior is consistent with the total phenolic content of the powders (Table 2), suggesting a correlation between antioxidant activity and the concentration of phenolic compounds in the products. Sarabandi et al. reported that powders obtained from the mixture of eggplant peel extracts with maltodextrin showed higher antioxidant activities than those obtained using gum Arabic as the encapsulating material [44].

### 3.4. Water Activity, Moisture Content, and Water Solubility of the Spray-Dried Powders

Table 3 shows the moisture content, water activity, and water solubility values of the MD, GA, and MD:GA spray-dried powders. All samples showed similar water activity values (aw~0.3), indicating that the encapsulating agents used did not affect this parameter. The GA powders displayed lower moisture content than the MD powders. In the case of MD:GA powders, intermediate moisture contents were observed between MD and GA powders. All samples exhibited high water solubility (>95%), which is suitable for their incorporation in aqueous food formulations. Several studies have reported that encapsulation via spray drying technology, using maltodextrin and gum Arabic as wall materials, leads to powders that are easily reconstituted at room temperature, with solubility values exceeding 90% [15,44].

### 3.5. Densities and Flow Properties of the Spray-Dried Powders

Table 4 presents the bulk density, tapped density, Hausner ratio, Carr index, and angle of repose of the MD, GA, and MD:GA spray-dried powders. The MD, MD:GA, and GA powders showed similar values of bulk density (*p* > 0.05), despite their variations in particle size and morphology. Previous studies have indicated that particles with larger sizes and a smooth, spherical appearances tend to have lower bulk densities [45,46]. Additionally, it has been reported that increasing the concentration of the carrier agent and decreasing the compressed air flow rate can lead to a decrease in bulk density. On the other hand, all samples were compacted after being subjected to vertical impacts, showing higher values of tapped density than bulk density. This behavior could be due to the presence of attractive and friction forces between the powder microparticles [34]. These values of bulk density and tapped density were lower than those obtained for freeze-dried Andean blueberry juice powders [9,10], which may be related to the high-water evaporation rates achieved at high temperatures, resulting in large particles with a porous structure. Similar results were reported by other authors when spray drying eggplant peel extract [44] and sumac extract [47].

Regarding the Hausner and Carr indices, no significant differences were found between the different encapsulating materials used, obtaining values of between 1.1 and 1.2 and between 13 and 18%, respectively. According to the literature, powders with Hausner ratios lower than 1.2 and Carr indices between 11 and 20% have good flowability and compressibility [34].

In general, the powders showed angle of repose values ranging from 22 and 35°, which is characteristic of materials with good flowability (Table 4) [34]. The MD powders showed better flowability than the GA powders (Table 4). In the case of MD:GA powders, the intermediate angle of repose values between MD and GA powders were obtained (Table 4). These findings are consistent with the SEM observations suggesting that GA and MD:GA powders with surface dents demonstrate lower flow properties compared to MD samples with a smooth surface [33]. Similar observations were reported by Sarabandi et al. in spray-dried apple juice and eggplant peel extract using a combination of various wall materials [44,48].

## 4. Conclusions

The use of maltodextrin, gum Arabic, and their combination as wall materials for the encapsulation of Andean blueberry juice via spray drying, allowed the obtaining of powders with desirable color characteristics in reddish tones, low moisture content (<5.4%) and water activity (a_w_ < 0.30), and favorable solubility in water (>95%) and flowability.

In particular, the combination of maltodextrin and gum Arabic as wall materials leads to powders with higher total polyphenol content and antioxidant activity compared to the samples produced using only a single encapsulating agent. Meanwhile, the highest total monomeric anthocyanin content was observed for the sample with only maltodextrin. The recovery percentages of phenolic compounds were above 80%, being higher than the recovery in the samples with the blend of wall materials (87%), while the recovery of monomeric anthocyanins was higher in the powders with only maltodextrin (96%). Overall, the results of this study suggest that spray-dried Andean blueberry juice powders have the potential to be used as a functional ingredient in the food industry.

## Figures and Tables

**Table 1:** Color attributes of the spray-dried Andean blueberry juice powders produced with maltodextrin (MD), gum Arabic (GA), or a combination of both materials (MD:GA).

Samples	a*	b*	L*	*h*	Chroma
GA	42.2 ± 1.6 ^a^	2.6 ± 0.2 ^a^	55.0 ± 1.8 ^b^	3.6 ± 0.2 ^a^	42.3 ± 1.6 ^a^
MD:GA	45.9 ± 0.4 ^b^	3.8 ± 0.3 ^b^	52.7 ± 2.5 ^b^	4.7 ± 0.3 ^b^	46.0 ± 0.4 ^b^
MD	45.6 ± 0.5 ^b^	6.1 ± 0.1 ^c^	47.5 ± 0.2 ^a^	7.6 ± 0.1 ^c^	46.0 ± 0.5 ^b^

Different superscripts in the same column indicate statistically significant differences (*p* < 0.05).

**Table 2:** Content and recovery percentage of total polyphenols and total monomeric anthocyanins, and DPPH scavenging activity of the spray-dried Andean blueberry juice powders produced with maltodextrin (MD), gum Arabic (GA), or a combination of both materials (MD:GA).

Samples	Total Polyphenols Content (mg GAE.g^−1^)	Total Polyphenols Recovery (%)	Total Monomeric Anthocyanins (mg C3G.g^−1^)	Total Monomeric Anthocyanins Recovery (%)	DPPH Scavenging Activity (mg GAE.g^−1^)
GA	5.17 ± 0.04 ^c^	81.5 ± 2.8 ^a^	0.70 ± 0.05 ^c^	80.8 ± 3.2 ^b^	2.14 ± 0.02 ^c^
MD:GA	5.70 ± 0.09 ^b^	87.2 ± 1.1 ^b^	0.78 ± 0.03 ^b^	83.6 ± 3.6 ^b^	2.49 ± 0.02 ^b^
MD	5.40 ± 0.03 ^a^	81.5 ± 0.5 ^a^	0.88 ± 0.02 ^a^	96.3 ± 1.7 ^a^	2.41 ± 0.02 ^a^

GAE: Gallic acid equivalents; C3G: cyanidin 3-glucoside equivalents. Different superscripts in the same column indicate statistically significant differences (*p* < 0.05).

**Table 3:** Water activity, moisture content, and water solubility of the spray-dried Andean blueberry juice powders produced with maltodextrin (MD), gum Arabic (GA), or a combination of both materials (MD:GA).

Samples	Water Activity (a_w_)	Moisture Content (%)	Water Solubility (%)
GA	0.30 ± 0.01 ^a^	4.7 ± 0.3 ^b^	96.4 ± 1.0 ^a,b^
MD:GA	0.29 ± 0.01 ^a^	5.1 ± 0.2 ^a,b^	96.7 ± 1.0 ^b^
MD	0.28 ± 0.02 ^a^	5.4 ± 0.3 ^a^	95.0 ± 0.3 ^a^

Different superscripts in the same column indicate statistically significant differences (*p* < 0.05).

**Table 4:** Densities and flow properties of the spray-dried Andean blueberry juice powders produced with maltodextrin (MD), gum Arabic (GA), or a combination of both materials (MD:GA).

Samples	Bulk Density (kg.m^−3^)	Tapped Density (kg.m^−3^)	Hausner Index	Carr Index (%)	Angle of Repose (°)
GA	329 ± 5.2 ^a^	404 ± 23 ^b^	1.2 ± 0.06 ^a^	16.3 ± 4.5 ^a^	34.5 ± 2.7 ^c^
MD:GA	323 ± 15 ^a^	389 ± 11 ^ab^	1.1 ± 0.04 ^a^	13.6 ± 2.9 ^a^	29.2 ± 3.3 ^b^
MD	302 ± 24 ^a^	353 ± 17 ^a^	1.2 ± 0.10 ^a^	17.6 ± 3.0 ^a^	22.9 ± 0.4 ^a^

Different superscripts in the same column indicate statistically significant differences (*p* < 0.05).

## Data Availability

The data that support the findings of this study are available on request from the corresponding author.
